# Uterine Arteriovenous Malformation With Raised Serum Beta-Human Chorionic Gonadotropin (β-hCG) Levels: A Diagnostic and Therapeutic Dilemma

**DOI:** 10.7759/cureus.64209

**Published:** 2024-07-10

**Authors:** Biswajit Sahoo, Saubhagya K Jena, Sourav K Mishra, Subarna Mitra, Arunprakash Pitchaimuthu, Manoj Nayak

**Affiliations:** 1 Radiology, All India Institute of Medical Sciences, Bhubaneswar, Bhubaneswar, IND; 2 Obstetrics and Gynecology, All India Institute of Medical Sciences, Bhubaneswar, Bhubaneswar, IND; 3 Medical Oncology, Advanced Medical Research Institute, Bhubaneswar, Bhubaneswar, IND

**Keywords:** interventional radiology, vaginal bleeding, uterine artery embolization, raised serum β-hcg levels, uterine arteriovenous malformation

## Abstract

Uterine arteriovenous malformations (AVM) are rare and usually present in women of reproductive age. Clinical presentation may overlap with early pregnancy, retained products of conception (RPOC), or gestational trophoblastic disease (GTD) if it occurs in a pregnant patient or the immediate postpartum period and becomes challenging to manage. Here, we present two cases of uterine AVM that presented with vaginal bleeding after miscarriages. In these cases, the presentation was vaginal bleeding with raised serum beta-human chorionic gonadotropin (β-hCG) levels. The uterine AVM was diagnosed with ultrasound and contrast-enhanced CT and subsequently managed with uterine artery embolization. Although rare, uterine AVM should be kept in the differentials in a premenopausal patient with abnormal vaginal bleeding and positive serum β-hCG levels. It should be differentiated from other common causes of vaginal bleeding with raised serum β-hCG levels, such as early pregnancy, GTD, and RPOC, as early diagnosis and proper treatment are crucial for favorable outcomes.

## Introduction

Uterine arteriovenous malformations (AVM) are rare and usually present in women of reproductive age. It is usually acquired in nature and follows events such as previous uterine surgery, delivery, therapeutic abortion, infection, gestational trophoblastic disease (GTD), and endometrial curettage [1,2]. A diagnosis of uterine AVM is confirmed based on radiological imaging or pathological examination and a negative serum beta-human chorionic gonadotropin (β-hCG) level [3,4]. However, uterine AVM with an elevated serum β-hCG level in patients with early pregnancy, GTD, and retained products of conception (RPOC) has been observed, which creates confusion [5]. Furthermore, a uterine AVM can be mistaken for GTD or RPOC on ultrasound (US) and color Doppler due to increased myometrial vascularity, and any attempt at evacuation by suction dilatation and curettage in these patients can cause life-threatening bleeding with an increase in morbidity and mortality. Therefore, it is imperative to differentiate uterine AVM with elevated serum β-hCG levels from other common causes of vaginal bleeding with raised serum β-hCG levels, such as early pregnancy, GTD, and RPOC, as the management varies. Clinically and radiologically, uterine AVM may overlap with early pregnancy, RPOC, or GTD if it occurs in a pregnant patient or the immediate postpartum period, and it is often missed due to its low incidence [6]. Here, we aim to describe the patient’s condition, the challenges encountered in diagnosis, and the effective and safe management of these rare cases of uterine AVM with elevated serum β-hCG levels.

## Case presentation

Case 1

**Figure 1:**
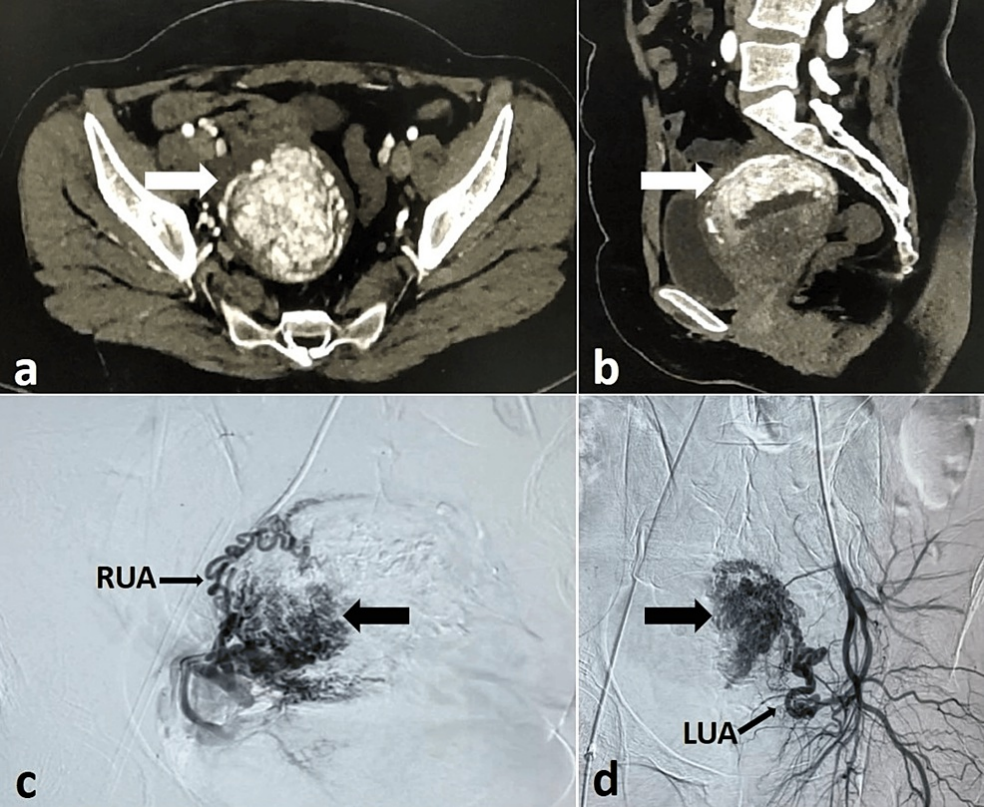
CT and pre-embolization DSA images of the patient with AVM CECT arterial phase axial (a) and sagittal (b) images showing an AVM (white arrows) involving the anterior myometrium. (c, d) Pre-embolization DSA images showing the low flow AVM (thick black arrows). AVM, arteriovenous malformation; CECT, contrast-enhanced CT; DSA, digital subtraction angiography; LUA, left uterine artery; RUA, right uterine artery

A 29-year-old female (G2P1L1A1) presented to our institution with a five-day history of profuse vaginal bleeding and abdominal pain. Her vitals were stable. She had a history of one cesarean section four years ago and a spontaneous abortion at 16 weeks of gestation five months ago, which was uneventful. Her serum β-hCG level was slightly elevated (296 mIU/mL). Despite medical management and vaginal packing, the bleeding persisted. The US revealed multiple tortuous and dilated vessels involving the fundus and anterior myometrium, consistent with AVM. Additionally, a large heterogeneous complex-echoic mass without significant vascularity on color Doppler was found within the endometrial cavity. No intrauterine gestational sac or fetal pole was identified. Subsequent contrast-enhanced CT (CECT) of the abdomen confirmed these findings (Figure 1a, 1b).

**Figure 2:**
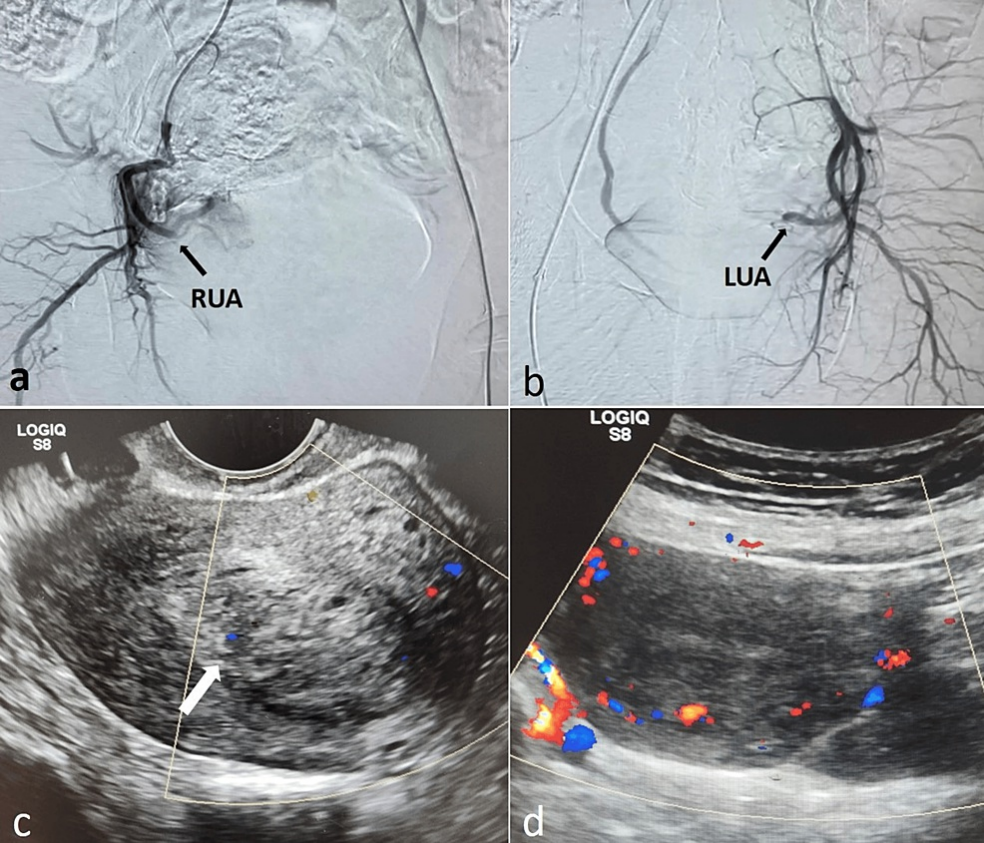
Post-embolization DSA and US images of the patient with AVM (a, b) Post-embolization DSA images showing non-opacification of the uterine AVM after embolization with NBCA/glue. Post-UAE: (c) Transvaginal US image on day 1 of intervention showing a bulky uterus with a heterogeneous complex-echoic mass within the endometrial cavity (white arrow); however, no abnormal vascularity was detected in the uterine wall on color Doppler. (d) Transabdominal US image after two months showing a normal-sized uterus with no endometrial mass or abnormal vascularity. AVM, arteriovenous malformation; DSA, digital subtraction angiography; LUA, left uterine artery; NBCA, n-butyl-2-cyanoacrylate; RUA, right uterine artery; UAE, uterine artery embolization; US, ultrasound

A diagnosis of uterine AVM with RPOC or GTD was established based on clinical, biochemical, and radiological findings. Dilatation and curettage were deemed unsafe due to the risk of life-threatening vaginal bleeding. After unsuccessful medical management, options for hysterectomy or uterine artery embolization (UAE) were discussed. The patient declined the hysterectomy but consented to UAE after consultation with interventional radiology. Digital subtraction angiography (DSA) of the uterine arteries revealed hypertrophied vessels with multiple tortuous channels and early venous drainage into the iliac veins, consistent with AVM (Figure 1c, 1d). Using a microcatheter (Progreat, Terumo, Japan), the AVM was embolized with a mixture of 30% n-butyl-2-cyanoacrylate (NBCA, Endocryl, Samarth Life Sciences Pvt. Ltd., India) and lipiodol (Guerbet, France) until flow stasis was achieved (Figure 2a, 2b).

On the first day post-embolization, color Doppler revealed no abnormal vascularity in the uterine wall (Figure 2c). Two months later, follow-up US showed a normal uterus with complete resolution of the uterine AVM and an empty endometrial cavity (Figure 2d). Her serum β-hCG levels were normal during the follow-up. By three months, the patient had resumed her menstrual cycles.

Case 2

**Figure 3:**
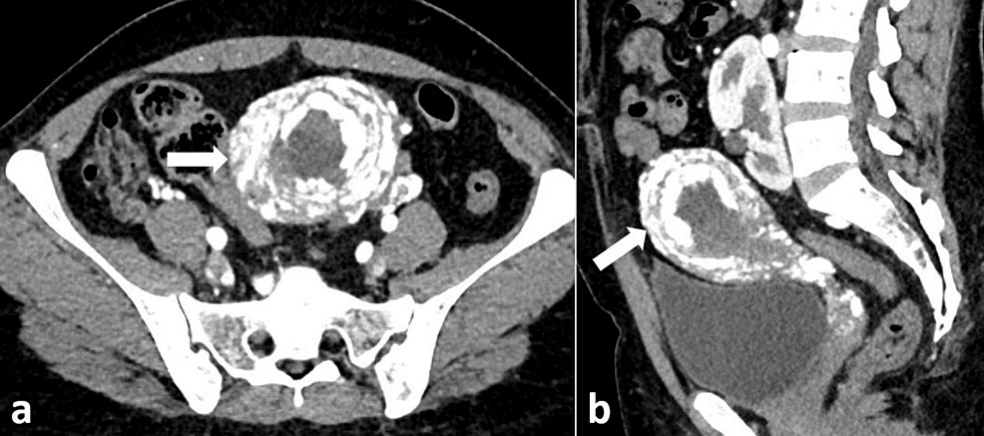
CT images of the patient with AVM CECT axial (a) and sagittal (b) arterial phase images show AVM (white arrows) involving the anterior and posterior myometrium with hypertrophied bilateral uterine arteries. The left kidney is ectopic in location. AVM, arteriovenous malformation; CECT, contrast-enhanced CT

A 34-year-old female (G3P1L1A2) presented to our emergency department with intermittent vaginal bleeding over five days. Her vitals were stable. She had a history of two previous spontaneous abortions, managed by suction and evacuation, three and two years ago, respectively. US and CECT showed AVM involving the anterior and posterior myometrium, with variable-sized anechoic cystic structures within the endometrial cavity (Figure 3a, 3b).

**Figure 4:**
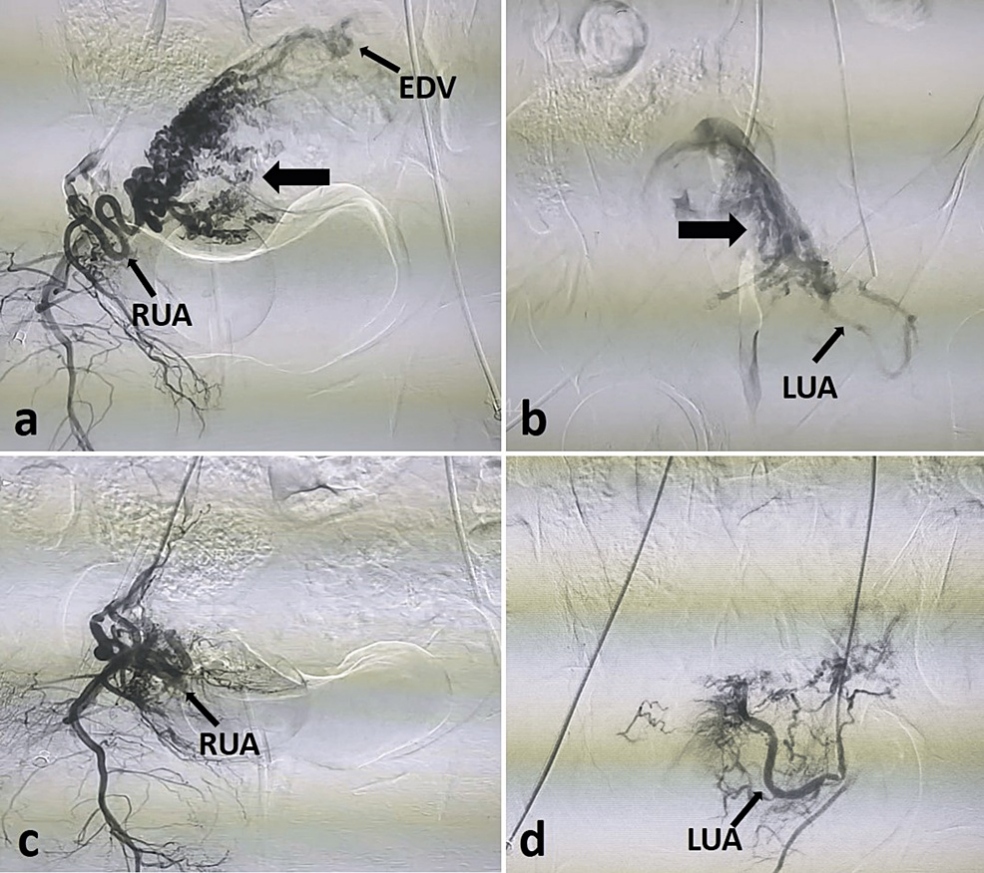
DSA images of the patient with AVM (a, b) Pre-embolization DSA images showing uterine AVM (thick black arrows) with arterial feeders from bilateral uterine arteries with EDV. (c, d) Post-embolization DSA images showing non-opacification of the uterine AVM after embolization with NBCA/glue. AVM, arteriovenous malformation; DSA, digital subtraction angiography; EDV, early draining veins; LUA, left uterine artery; NBCA, n-butyl-2-cyanoacrylate; RUA, right uterine artery

She tested positive on a urine pregnancy test with a slightly elevated serum β-hCG level (304 mIU/mL). Based on biochemical, clinical, and radiological findings, a presumptive diagnosis of uterine AVM with GTD was made. The patient underwent transarterial embolization of bilateral uterine arteries using NBCA (Endocryl, Samarth Life Sciences) mixed with lipiodol (Guerbet) (Figure 4a-4d).

**Figure 5:**
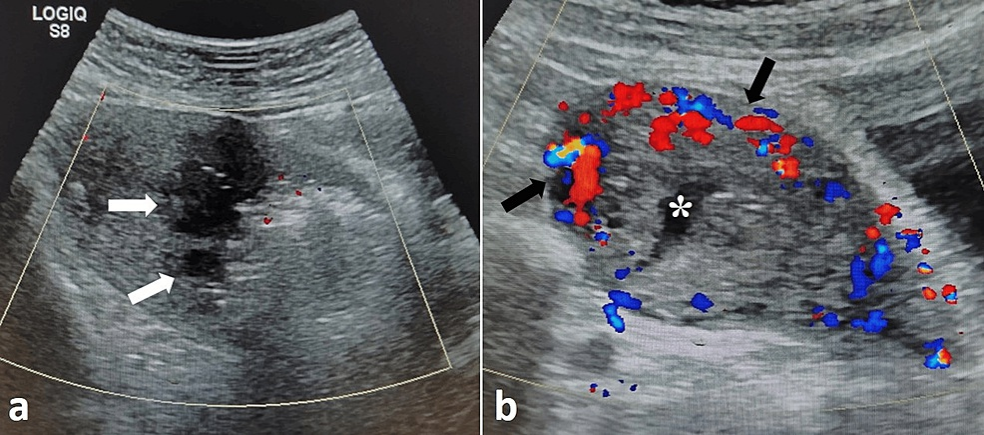
US images of the patient with AVM Post-UAE: (a) Transabdominal US image on day 1 of intervention showing bulky uterus with few variable-sized anechoic cystic structures within the endometrial cavity (white arrows); however, no abnormal vascularity was detected in the uterine wall on Color Doppler. (b) Transabdominal US image after three months showing a normal-sized uterus with normal uterine vessels (black arrows) and minimal endometrial collection (asterisk). AVM, arteriovenous malformation; UAE, uterine artery embolization; US, ultrasound

On day 1 post-embolization, no abnormal vascularity was detected in the uterine wall on color Doppler (Figure 5a).

After two months post-embolization, the patient resumed her menstrual cycle. At three months, follow-up US revealed a normal-sized uterus without abnormal vascular lesions or vaginal bleeding episodes (Figure 5b). Her serum β-hCG levels remained normal during the follow-up.

## Discussion

Uterine AVMs are rare and are usually acquired in nature and follow events such as previous uterine surgery, delivery, therapeutic abortion, infection, GTD, and endometrial curettage [1,2]. A confirmatory diagnosis of uterine AVM is based on radiological imaging or pathological examination and a negative serum β-hCG level [3,4]. Usually, uterine AVM accompanies a negative serum β-hCG level; however, uterine AVM with an elevated serum β-hCG level in patients with early pregnancy, GTD, and RPOC has been observed. Darlow et al. have described uterine AVM with elevated serum β-hCG levels in four patients [5]. The chief clinical symptom of uterine AVM is recurrent and profuse vaginal bleeding, which may be life-threatening [6,7]. US with color Doppler is the preferred initial imaging modality for evaluating uterine AVM. On the gray-scale US, the endometrium appears normal, with an inhomogeneous myometrium containing multiple tortuous and dilated anechoic structures or cystic spaces. On color Doppler, these anechoic structures or cystic spaces appear as tangles of vessels in the myometrium with multidirectional turbulent flow, generating a mosaic pattern of colors [3]. Still, this pattern on the US and color Doppler can sometimes be seen in GTD and RPOC due to increased myometrial vascularity, leading to confusion with uterine AVM. Therefore, CECT, MRI, or DSA should be considered for diagnosing uterine AVM in cases of any diagnostic dilemma, primarily in cases with raised serum β-hCG levels [8]. The lesion will show bilateral tortuous and dilated feeding uterine arteries and markedly dilated venous structures with early contrast filling on angiography.

For management purposes, uterine AVM with elevated serum β-hCG levels should be differentiated from other common causes of vaginal bleeding with raised serum β-hCG levels, such as early pregnancy, GTD, and RPOC; the management varies. The management of GTD includes evacuation by suction dilatation and curettage or hysterectomy, followed by serum β-hCG level surveillance. For RPOCs, uterotonic medications and surgical interventions such as suction dilation, curettage, hysteroscopic removal, and expectant management are available. Based on the US, CECT, and raised serum β-hCG levels, we presumed that our patients had uterine AVM with associated RPOC or GTD. A curettage or surgical biopsy was avoided in our patients due to the possibility of life-threatening vaginal bleeding. The management of uterine AVMs ranges from a conservative approach, such as observation or medical management (single or combination chemotherapy), to invasive procedures, such as UAE or hysterectomy [9,10]. For patients presenting with vaginal spotting or nonspecific symptoms, weekly clinical follow-up is performed, including US and serum β-hCG level measurements. Chemotherapy is considered for cases with sustained or elevated serum β-hCG levels after two consecutive clinical follow-ups [10]. A UAE can be considered when vascular lesions fail to respond to chemotherapy. If there is unrestrained, uncontrolled vaginal bleeding with stable vital signs, UAE is performed. An emergency hysterectomy is performed if the patient has unstable vital signs because of profuse vaginal bleeding [9].

Although the probability of GTD was low in our patients due to lower serum β-hCG levels than the diagnostic criteria, GTD should be considered in the differential diagnosis. The treatment options for uterine AVM depend on the clinical status of the patient, the severity and amount of vaginal bleeding, and the patient’s preference to conserve fertility [3]. UAE is generally accepted as the best possible treatment method for symptomatic uterine AVM due to lower complication rates, minimal invasiveness, and preserving fertility [11]. After treatment, US and serum β-hCG level measurements should be done regularly to check for recurrence.

## Conclusions

While rare, uterine AVM should be considered in the differential diagnosis for premenopausal patients presenting with abnormal vaginal bleeding, positive serum β-hCG levels, and conditions such as early pregnancy, GTD, or RPOC. Clinically and radiologically, uterine AVM may overlap with early pregnancy, RPOC, or GTD if it occurs in a pregnant patient or in the immediate postpartum period. A Doppler examination should be performed in these patients to diagnose uterine AVM; however, CECT, MRI, or DSA can be performed in cases of a diagnostic dilemma, primarily in cases with raised serum β-hCG levels. It is essential to differentiate uterine AVM with elevated serum β-hCG levels from other common causes of vaginal bleeding with raised serum β-hCG levels, such as early pregnancy, GTD, and RPOC, as precise diagnosis and appropriate treatment are crucial for favorable outcomes.
